# From Leaves to Litter: Use of Anthropogenic Nesting Materials in Hibernation Nests of the European Hedgehog

**DOI:** 10.1002/ece3.72844

**Published:** 2026-01-02

**Authors:** Katie Crawford, Christine E. Beardsworth, Davina L. Hill, Ross MacLeod, Julia Nowack

**Affiliations:** ^1^ School of Biological and Environmental Sciences Liverpool John Moores University Liverpool UK; ^2^ School of Biodiversity, One Health and Veterinary Medicine University of Glasgow Glasgow UK; ^3^ School of Animal, Plant and Environmental Sciences University of the Witwatersrand Johannesburg South Africa

**Keywords:** behavioral adaptation, climate change, hibernation nests, nesting behavior, plastic, urbanization

## Abstract

Urbanization and human population growth have significantly increased the presence of anthropogenic materials in natural environments, prompting growing interest in how wildlife may be adapting to these changes. One such behavioral response is the incorporation of anthropogenic materials into animal nests, a phenomenon that has raised concerns due to its potential harmful effects, such as entanglement or ingestion. While this behavior has been documented widely in birds, it remains underreported in other taxa, partly due to the difficulty of locating nests. In this study, we describe multiple instances of anthropogenic materials (including plastic) being incorporated into the hibernation nests of European hedgehogs, *Erinaceus europaeus* . Four nests were dissected, of which two nests contained anthropogenic materials, including a plastic bag, foil and expanded polystyrene. These findings suggest that hedgehogs may opportunistically use available anthropogenic materials in nest construction, potentially as a response to urban environments. Our findings help broaden the understanding of mammalian responses to urbanization and emphasize the need to investigate whether the incorporation of these materials is likely to be harmful or adaptive to hedgehogs and for mammals generally.

## Graphical abstract

This article presents the first detailed description of anthropogenic materials, including plastics, being incorporated into the hibernation nests of European hedgehogs ( *Erinaceus europaeus* ). While such behavior is well documented in birds, it has been underreported in terrestrial mammals—likely due to difficulties accessing mammalian nests—highlighting both potential risks (entanglement, ingestion) and possible adaptive responses to urbanization. These findings broaden the understanding of mammalian nesting behavior in human‐altered environments.

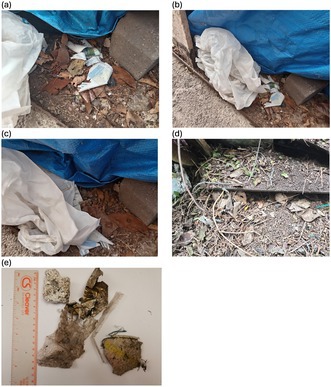

## 1 Introduction

Urbanization has steadily increased alongside global population growth (Sun et al. 2020), leading to profound impacts on natural habitats. These include habitat destruction and fragmentation, as well as increased levels of light, noise, and chemical pollution (Barber et al. 2010; Grimm et al. 2008; Navara and Nelson 2007). While such changes are generally detrimental to biodiversity, some species have shown the capacity to adapt and even thrive in urban environments (McKinney 2006). Understanding how species respond and adapt to urbanization is crucial for informing conservation efforts, particularly for species that are vulnerable within urban environments.

Urbanization is associated with the degradation and fragmentation of natural habitats (Liu et al. 2016), which can reduce the availability of resources for species (Simkin et al. 2022), including the materials needed for nest building. The incorporation of plastic and other anthropogenic materials into animal nests has been extensively described in birds (Jagiello et al. 2019; Luna et al. 2024; Townsend and Barker 2014; Votier et al. 2011). Materials found have included objects such as confectionery wrappers, cigarette butts, and plastic strings, with materials varying by habitat (Jagiello et al. 2019). These materials are especially prevalent in urban environments where their diversity reflects higher levels of plastic waste accumulation (Brglez et al. 2024). However, plastic can also be found in remote locations, such as the Arctic (Bergmann et al. 2022) and the Tibetan Plateau (Wang et al. 2022). A global review based on 25 articles and covering 24 bird species found that 31% of 10,790 nests contained anthropogenic material (Jagiello et al. 2019). The presence of these materials has been shown to incur both costs and benefits. For example, microplastic ingestion by predators, likely as a result of trophic transfer from prey, has been documented in barn owls, *Tyto alba*, (Pietrelli et al. 2024) with negative impacts including biochemical alterations predictive of oxidative stress and body biomass reduction (de Silva Souza et al. 2022). Cases of entanglement, especially in nestlings, have been widely reported in birds (Heinze et al. 2025; Townsend and Barker 2014). On the other hand, potential benefits include ectoparasite reduction through incorporating materials such as cigarette butts with chemical compounds that repel parasites and structural reinforcement of nests (Jagiello, Reynolds, et al. 2023).

Despite this growing body of avian research, there is very little information on non‐bird nest construction, including in mammals. As a result, we lack information on how widespread anthropogenic material incorporation is across these taxa, which materials they select, and how those choices affect fitness.

The European hedgehog, *Erinaceus europaeus*, is one such nest building mammal commonly found in suburban and urban habitats (Korslund et al. 2024). Hedgehogs typically build three types of nests: day nests for summer shelter, breeding nests where females raise young, and hibernation nests used during the hibernation season when individuals enter torpor and remain in a state of extended inactivity (Reeve 1994). The latter are normally used for longer periods of time than summer nests (Gago et al. 2023). Hedgehog hibernation nests in the UK, where most studies have taken place, are typically composed of grass and leaves packed together and often occur within areas of structural support, such as underneath bramble, *Rubus fruticosus agg*. (Morris 1973). Hibernation nests are typically constructed in woodland patches or other natural areas, even when suburban areas are used during the active season (Korslund et al. 2024; Rautio et al. 2014). In both urban and rural landscapes, hedgehogs select nesting locations within hedgerows and bushes (Jensen 2004; Korslund et al. 2024).

Hedgehog populations in Britain are decreasing in rural areas but stabilising or possibly increasing in urban areas (Wembridge et al. 2022). This difference is thought to result from a combination of factors, including supplementary feeding regimes and decreased predation risk by badgers, *Meles meles* . Increased availability of shelter (such as shrubbery) may also provide more hibernation nesting sites compared to intensively managed agricultural landscapes (Hubert et al. 2011). While the presence of anthropogenic materials in hedgehog nests was noted in Rautio et al. (2014), no detailed descriptions were provided for hibernation nests specifically.

Here, we document the discovery of anthropogenic materials in hibernation nests of European hedgehogs living in suburban habitats in the UK. As hedgehogs increasingly frequent suburban and urban environments, they are more likely to encounter anthropogenic materials, which they might use as nesting material, despite the potential hazards these materials can pose. Thus, we suggest that, as in birds, the increased prevalence of hedgehogs in urban environments may lead to an increase in the use of plastics and other anthropogenic substrates as nesting material, and that this behavior may incur both benefits and risks, including ingestion and entanglement as described above.

## 2 Methods

We collected data on nest site use during hibernation of 10 individual hedgehogs (4 male, 6 female) across 6 different locations within Merseyside in the Northwest of England (Lat: 53.407485, Long: −2.9882813). Survey locations ranged from more urbanized/residential areas to more suburban or semi‐rural green spaces (such as parks and a disused golf course, see Table 2).

To find and capture hedgehogs, we conducted night surveys at various locations across Merseyside. As part of another study, 10 individuals captured between 1st August and 1st November 2024 were equipped with temperature‐sensitive VHF radio transmitters (TW‐3 single cell tag with thermistor, Lotek, UK) that allowed us to detect hibernation start and end dates. These were attached to the back of the individual, with the thermal attachment touching the skin. Spines were clipped, and the tag glued on using a combination of epoxy resin for attaching to spines and bonding cement (Torbot, Torbot Group Inc., US) to affix it nearer to the skin (Crawford et al. 2025). All tags weighed approximately 12 g, which is < 2% of the animal's body mass (adult body mass 700–1000 g).

We used the tags to find and monitor hibernation nest site use of 10 individuals during their hibernation period, for a total of 207 days (10th September 2024–4th April 2025). Individuals were radio‐tracked and located every 1–3 days, and readings of skin temperature were remotely taken over the course of the winter hibernation period to identify the start and end of hibernation for each individual. During this data collection period, we identified the location and type of nests used as well as the duration of use and frequency of nest site changes. We noted the microhabitat (e.g., within a log pile, in a garden, under bramble) and recorded each nest location (latitude and longitude) using smartphone GPS (Huawei P30 Pro) and the Google Maps app (Google 2025). Most nests were too deeply hidden to see without disturbing the animal, but for some nests, we were able to visibly assess the primary material of the nest (e.g., leaves, grass, etc.).

In total, four nests were fully or partially dismantled and the materials found in them visually identified in the field. In March 2025, two of these nests from residential urban/suburban areas were dissected. One dissection was conducted to retrieve a detached radio tag (hedgehog #385, female), which required partially dissecting the nest and removing material from the hidden nest to access the device. The second was prompted by prior observations of the individual in the nest in September 2024, during which anthropogenic materials had been noted. The nest was subsequently dismantled in March 2025 when the individual was not present (hedgehog ID #366, male). Another hedgehog (#379, female) was briefly uncovered from a nest in November 2024 to ascertain if its tag had detached. This nest was only partially dismantled. The fourth hibernation nest was used by a male hedgehog (hedgehog #376) and located on a disused golf course (rural habitat), and was dissected in February 2025 to retrieve a radio tag that had already been abandoned by the animal. For ethical reasons, nests that were partially dismantled were subsequently left in situ and all other known nests were left undisturbed as they were occupied, and hibernation nests can be reused (Crawford et al. 2025).

To evaluate how urban these nest sites were, we calculated the percentage of urban and suburban land within 1 km of each of the nests using the 2023 land cover map (resolution 10m^2^) from the NERC EDS Environmental Information Data Centre (Morton et al. 2024). These data use satellite imagery to classify urban and suburban land, with urban land constituting a high proportion of sealed surface and suburban comprising a mix of urban land and vegetated land.

## 3 Results

### 3.1 Nest Site Use During Hibernation

Across all 10 hedgehogs, 24 separate nests were used, with individuals occupying 1–3 nests each (Table 1). Mean nest‐use duration was 41.4 ± 40.5 (SD) days. Individual nest‐use durations ranged from 1 to 128 days. Three individuals (#332, #366, and #385) occupied the same nest site more than once over the duration of their hibernation.

**TABLE 1:** Hibernation duration and nest site use for radio‐tracked hedgehogs in this study (Merseyside, UK) across 2024–2025.

Hedgehog ID	Hibernation start date	Hibernation end date	Hibernation length (days)	Days recorded	Sex	No. of nest sites
#366	23/09/2024	20/02/2025	151	151	M	3
#390	18/11/2024	04/04/2025	138	138	F	2
#370	07/10/2024	13/02/2025	130	130	M	3
#332	10/09/2024	27/02/2025	171	171	M	3
#375	04/12/2024	20/03/2025	107	107	F	3
#379	15/11/2024	04/03/2025	110	110	F	2
#391	04/11/2024	20/03/2025	137	137	F	2
#371	15/11/2024	NA, tag lost 25/11/24	NA	11	F	1
#385	30/09/2024	NA, tag lost 30/11/24	NA	62	F	3
#376	23/10/2024	NA, tag lost 25/12/24	NA	64	M	2

### 3.2 Anthropogenic Material in Nests

**FIGURE 1:**
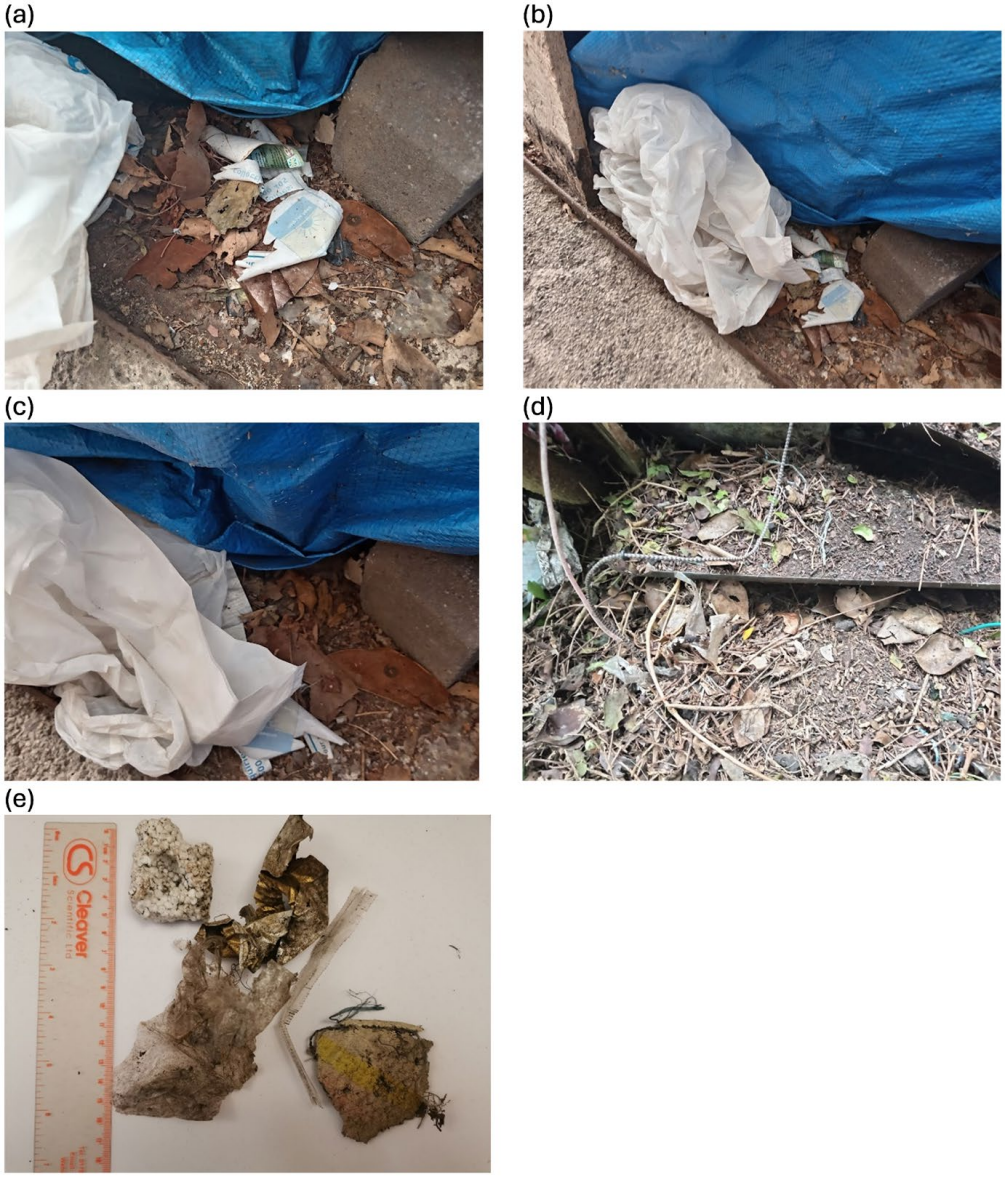
Examples of anthropogenic materials within hedgehog nests observed during the study: (a) the nest of individual #366 primarily comprising a plastic bag, (b) stray miscellaneous plastic within the nest of #366, (c) duct tape within the nest of #366 (behind plastic bag), (d) location of the nest of #385 underneath a metal sheet, surrounded by rope and plastic, (e) anthropogenic material pulled from the nest of #385 (comprising foil, plastic wrappers and expanded polystyrene).

Of the four dissected nest sites, anthropogenic material was found in two nests:

Hedgehog #366 nested underneath a disused car with plywood covering the sides of the car, in a location with the highest proportion of urban land out of the four locations (Table 2). The nest contained various anthropogenic materials, including a plastic bag, duct tape, and several pieces of plastic (Figure 1a–c, Table 2). Leaves were present on the ground, but these were not directly incorporated into the nest. In contrast, hedgehog #385 nested under metal sheeting in a suburban garden within a shaded area of trees and shrubs (Figure 1d,e, Table 2) with rope encircling the nest and other anthropogenic materials, including foil and expanded polystyrene incorporated into the nest. This nest also contained natural materials, including leaves and dried grasses, that were woven in with the anthropogenic materials. It was noted that the garden in which this hedgehog nested (second‐highest urban land cover, Table 2) was littered with anthropogenic waste materials. These nests were used for 20 and 99 days, respectively.

**TABLE 2:** Descriptions of hedgehog nests, including periods of use, materials discovered, and habitat type.

Hedgehog ID	Sex	Start date of nest use	End date of nest use	No. days occupied	Anthropogenic materials discovered	Percentage of urban land within 1 km	Percentage of urban and suburban land within 1 km	Habitat
#366	M	18/09/2024	07/10/2024	20	Plastic bag, duct tape, various plastic pieces	33.4%	96.2%	Residential area (under car)
#385	F	09/10/2024	15/01/2025	99	Rope, foil packet, plastic straw packet, plastic residue	15.1%	55.4%	Residential area, garden
#376	M	26/10/2024	26/12/2024	61	None	1.67%	14.7%	Disused golf course (semi‐rural)
#379	F	15/11/2024	21/11/2024	7	None	5.5%	87%	Residential area, hedgehog box in garage

Of the remaining two hedgehogs located in areas with significantly lower urban land cover (Table 2), #376 had a nest comprising woven grass and leaves, situated within a small patch of scrub adjacent to a woodland within a broadly semi‐rural area. No anthropogenic material was found in this nest. Similarly, the nest of #379 was primarily made of woven grass and straw and did not contain anthropogenic materials. This female nested in a suburban residential area within a wooden hedgehog nest box in a garage (Table 2). These nests were used for 61 and 7 days, respectively.

## 4 Discussion

This study provides the first detailed descriptions, to our knowledge, of anthropogenic materials being used within hedgehog hibernation nests. Anthropogenic materials were found in two nests in suburban/urban locations. Both nests were built within metal structures and contained a variety of waste materials. Rautio et al. (2014) previously noted that anthropogenic material was present in 6% of hedgehog nests but did not catalogue the types of anthropogenic materials found nor distinguish between nest types (e.g., day nests vs. hibernacula). In our study, the two nests containing anthropogenic material constitute a small proportion of all nests used by the 10 individuals tracked over the winter period. However, we only had cause to dissect 4 nests, and 50% of these nests contained anthropogenic material. Given that two out of the four individuals for which nests were dissected lost their radio tags during the investigation (of which one had a nest containing anthropogenic material and one did not), we have no information on the number of nests these two individuals made after they lost their tags. However, hedgehog #366, whose radio tag remained attached throughout (and whose nest primarily consisted of a plastic bag), used only one other nest site for the duration of hibernation. We do not know whether this other nest also contained anthropogenic materials. Individual #379 also retained its tag and did not reuse the hedgehog nest box site at a later stage of hibernation, although the nest had been used prior to hibernation onset. These observations indicate that anthropogenic materials can be incorporated into hibernation nests within suburban and urban environments and may provide possible functional implications.

Given that hedgehogs are often found in suburban and urban areas where natural nest‐building resources are scarcer than in natural environments, such as a mixture of woodland and fields, the incorporation of anthropogenic materials may serve to compensate for the limited availability of natural resources. This is supported by the two nests with anthropogenic material having the highest percentage of urban land cover (> 15%) within 1 km. Urban and suburban land within 1 km varied between individuals, with the nest of #366 having the highest percentage of both urban and suburban land (96.2%). This individual had a nest almost exclusively composed of anthropogenic materials. Both nests were occupied for several days at the onset of hibernation by these individuals, suggesting that nest conditions were functional in early winter. Whether such materials are used opportunistically or selected for their functional properties requires further investigation. In the Chinese bulbul *Pycnonotus sinensis* , researchers found that the proportion of anthropogenic materials in their nests increased with urbanization score (Wang et al. 2009). Similarly, a study on two populations of the white stork, *Ciconia ciconia*, revealed that the amount of anthropogenic material incorporated into nests was positively associated with the human footprint index in a Spanish population, whereas no relationship was found in a Polish population, which the authors attributed to a preference for more rural nest sites in this area (Jagiello, Dylewski, et al. 2023). A similar pattern may occur in European hedgehogs, particularly in environments where natural nesting materials may be less abundant. Incorporation of plastics and other anthropogenic materials into nests has been studied primarily in birds. Plastic nest boxes have been shown to create hotter, drier environments for birds (Noel et al. 2023). One of the hedgehogs used a man‐made nest box with no anthropogenic material within for part of the hibernation period, and studies have shown hedgehogs using nest boxes in both the active season and hibernation (Gazzard and Baker 2022), indicating that artificial refugia may provide suitable conditions for hibernating animals. A study indicated that bird nests that incorporate metal can become hotter at warm temperatures and cooler under cool conditions than wooden nest structures (Imlay et al. 2019). Therefore, metal could aid in creating a slightly cooler microclimate for hibernating hedgehogs during winter, allowing longer torpor bout durations and thus saving energy (Geiser and Ruf 2023). Thermal properties differ between materials and some anthropogenic materials could have particularly useful properties for nest building. For example, substances such as foam have been hypothesized to provide enhanced insulation (Jagiello, Reynolds, et al. 2023). These altered thermal properties may offer advantages to nest inhabitants; for instance, offspring in bird nests containing foam may be more easily kept within optimal temperature ranges (Jagiello, Reynolds, et al. 2023). Hedgehogs may likewise benefit from thermal regulation in breeding nests, which could support offspring development. Breeding females have less variable body temperatures than non‐breeding females (Fowler 1988), suggesting a greater requirement for stable temperatures in their breeding nests. Such thermal stability may also be important in hibernation and day nests for individual survival. It is also possible that these materials influence conditions beyond temperature, such as humidity. Expanded polystyrene, which was discovered in the nest of hedgehog #385, is both a thermal insulator commonly used in buildings and semi‐permeable to water vapor and therefore may provide stable internal conditions in terms of both heat and humidity for nesting (Kumar et al. 2020). In the hazel dormouse, *Muscardinus avellanarius*, low variability in humidity and shade levels are important condition for hibernation nest site selection (Findlay‐Robinson and Hill 2024). It is likely that hedgehogs require similarly stable conditions for hibernation nesting sites. Furthermore, anthropogenic materials in nests might have other potential benefits, such as increasing the stability of the nest structure. The use of plastic in bird nests can improve structural stability (Antczak et al. 2010) and even reduce parasite load in certain instances (Suárez‐Rodríguez et al. 2013). On the other hand, the incorporation of anthropogenic materials into nests might also have negative consequences for animal welfare. The presence of plastic within nests is well documented to cause mortality in birds (Janic et al. 2023; Townsend and Barker 2014; Votier et al. 2011), while entanglement is a frequent cause of hedgehog admissions to rescue centers (Thrift et al. 2022). Therefore, incorporating plastic materials into nests could pose a significant risk of entanglement for hedgehogs. These plastics could also pose a risk through ingestion, as studies have indicated a high prevalence of plastic in the feces of hedgehogs and other mammals (Thrift et al. 2022).

The nests identified in this study were investigated opportunistically as part of a larger project and were only disturbed when there was a clear justification for doing so. This resulted in a small sample size, limiting our ability to make conclusions about the frequency of this occurrence. Further observations are needed to understand the range of materials hedgehogs may use within urban environments and whether the types of anthropogenic materials incorporated into nests affect the nest microclimate and, in turn, hibernation success. In this study, nest materials were only identified visually in the field; subsequent analysis would provide more information on the proportions of different anthropogenic materials in nests. It also remains unclear whether hedgehogs actively select anthropogenic materials for their functional properties or incorporate them incidentally due to reduced availability of natural alternatives. Exploring the drivers of this behavior would improve understanding of the potential fitness consequences. Future studies could address this by comparing local material availability with material use within the nest, or by experimentally offering hedgehogs both natural and artificial nest materials to test for selection. Nevertheless, the presence of anthropogenic materials in hedgehog hibernation nests highlights a potential shift in nesting behavior within an urban context. This warrants further investigation to assess both the ecological consequences and the welfare implications for hedgehogs in these areas.

## Author Contributions

**Katie Crawford:** conceptualization (equal), data curation (lead), formal analysis (lead), investigation (lead), writing – original draft (lead), writing – review and editing (equal). **Christine E. Beardsworth:** conceptualization (equal), supervision (equal), writing – review and editing (equal). **Davina L. Hill:** conceptualization (equal), supervision (equal), writing – review and editing (equal). **Ross MacLeod:** conceptualization (equal), supervision (equal), writing – review and editing (equal). **Julia Nowack:** conceptualization (equal), data curation (supporting), formal analysis (supporting), funding acquisition (lead), investigation (supporting), methodology (lead), project administration (lead), resources (lead), supervision (lead), writing – review and editing (equal).

## Funding

The project was possible due to financial support from the School of Biological and Environmental Sciences at Liverpool John Moores University. The project was also funded by the Wild Animal Initiative (grant number: SG23‐017).

## Ethics Statement

Ethical approval for the study was obtained from Liverpool John Moores University (licence number: JN_KC/2024‐5) and a Licence issued by Natural England in order to carry out the study methods (licence number: 2022‐61869‐SCI‐SCI‐3).

## Conflicts of Interest

The authors declare no conflicts of interest.

## Data Availability

Data are accessible through figshare: https://figshare.com/s/0748fbac5f849b68a056.
